# Memory and thinking problems that aging Latinos in New York City would bring to a doctor’s attention

**DOI:** 10.1371/journal.pone.0349635

**Published:** 2026-05-20

**Authors:** Karolynn Siegel, María Cabán, Evelyn Tran, Alicia Meng, John B. Wetmore, Ruth Ottman

**Affiliations:** 1 Department of Sociomedical Sciences, Mailman School of Public Health, Columbia University New York, New York, United States of America; 2 Gertrude H. Sergievsky Center, Columbia University Irving Medical Center, New York, New York, United States of America; 3 Department of Epidemiology, Columbia University Mailman School of Public Health, New York, New York, United States of America; 4 Department of Neurology, Columbia University Irving Medical Center, New York, New York, United States of America; 5 Division of Translational Epidemiology and Mental Health Equity, New York State Psychiatric Institute, New York, New York, United States of America; University of Haifa, ISRAEL

## Abstract

The number of individuals with Alzheimer’s disease will grow dramatically in the coming decades. Early diagnosis benefits patients, caregivers and society, but depends heavily on afflicted individuals or their family members recognizing early symptoms as possible indications of a medical problem and seeking medical care. To examine the kinds of memory or thinking problems, Latinos ages 40–64 would seek medical care for, we analyzed data from 161 participants in a community-based study in northern Manhattan of the impact of receiving information about one’s risk of developing late-onset Alzheimer’s disease. Participants were asked whether experiencing each of 5 different memory or thinking problems multiple times over 2–3 months would make them seek medical care. Participants often offer a benign or normalizing attributions for symptoms. Disorientation was the most frequently endorsed problem. Considerations found to be associated with an inclination or disinclination to want to see a doctor about a symptom were identified. A better understanding of what Latinos would consider in deciding whether or not to bring different memory problems to a doctor’s attention can help guide the development of educational interventions to encourage help-seeking and facilitate earlier diagnosis.

## Introduction

In the US, an estimated 6.7 million people aged 65 or older are currently living with Alzheimer’s disease (AD). It is projected that this number will reach 12.7 million in 2050 [1], of which an estimate 2.6 million will be Latinos [2]. It has been noted that, “As the United States becomes a majority-minority nation by 2050, increases in the number of non-Hispanic whites with ADRD [Alzheimer’s disease and related dementias] will begin to plateau around 2030 while the number in minority populations will continue to grow, particularly among Hispanics” [3].

Due to the high costs of caring for individuals with AD, the US and other countries have mounted national strategies to address the disease and dementia more broadly [4]. A principal goal of these efforts is to achieve an early diagnosis, which can carry important economic and quality of life benefits, especially as new interventions become available, such as amyloid-lowering drugs [5]. Early diagnosis can give families of persons with AD more time to learn about the illness, prepare for changes that will come with disease progression, and become linked to supportive resources [6–8]. Yet research has documented often long delays from observation of the first signs or symptoms of AD until diagnosis [9]. This is especially true for minority older adults, including Latinos, who are more likely to experience a missed or delayed diagnosis for dementia than their non-Hispanic White peers [10–13]. In a study of almost 4,000 participants age 70 and above with probable dementia in the Health and Retirement Study (HRS) with linked Centers for Medicare & Medicaid Services (CMS) claims, Lin et al. [10] found 18% of their participants experienced a delayed dementia diagnosis. Of these, the diagnosis delay was 11% longer for non-Hispanic Blacks and 40% longer for Hispanics than for non-Hispanic Whites. When a delay occurred, it averaged 34.6 months for non-Hispanic Blacks and 43.8 months for Hispanics, compared to 31.2 months for non-Hispanic Whites. One possible explanation for this disparity is that minorities may be less inclined to see a provider about symptoms. A recent Alzheimer’s Association study reported that “… Hispanics, Black and Native Americans were twice as likely as White Americans to say they would not see a doctor if they were experiencing thinking or memory problems” [14].

As has been noted, “Currently, Alzheimer’s diagnosis in the primary care setting has been dependent mainly on clinical suspicion based on the patient’s or caregiver’s concerns rather than the use of assessment tools and is often prone to missed or delayed diagnoses” [15]. That is, in the absence of routine screening of older adults for cognitive impairments, early diagnosis largely depends on recognition of symptoms as possible signs of a medical condition and bringing them to the attention of a provider. However, many adults who experience symptoms do not consult their physician about them. A nationally representative survey of adults 45 and older found that of those reporting subjective cognitive decline (i.e., self-reported worsening or more frequent confusion and memory loss), fewer than half (46%) discussed these symptoms with a healthcare professional [16]. Other studies of mostly elderly individuals with memory complaints have found that only approximately a quarter or less had brought them to a physician’s attention [17–19].

A number of systematic reviews of the literature on formal and informal help seeking among people with such cognitive problem or dementia have been carried out [20–22]. The factors that have been found to be commonly associated with help-seeking have been: recognizing the symptoms as a problem, greater prior knowledge of the disease’s symptoms, support and assistance from one’s informal network in help-seeking decisions, greater perceived severity of the problem and its consequences, having a close family member with AD, valuing early detection, having a family history of dementia, believing one’s memory was worse than peers, and holding positive attitudes toward health care providers. While the factors found to be commonly associated with refraining from seeking help were normalizing cognitive problems and attributing them to psychosocial causes, other factors included fear, lack of knowledge, stigma, not perceiving a benefit to disclosing cognitive problems, and lack of informal support.

Not all research on professional help-seeking for cognitive problems has focused on individuals already experiencing cognitive difficulties or dementia symptoms. Some have presented convenience samples of adults with hypothetical scenarios or vignettes of individuals with cognitive symptoms to investigate the actions, if any, that they felt should be taken --including a consultation with a physician [23–26]. Berwald et al., [23] recruited an initial socioeconomically diverse sample of Black African and Caribbean adults (18 and older) from community groups followed by snowball sampling. Participants were read a case vignette about a 70-year-old African or Caribbean woman experiencing memory problems. They were then asked how they would help if a relative or close friend experiencing similar problems, and whether they would encourage them to seek help and from what source. Many felt that forgetfulness was not a severe enough problem to talk to a medical provider about. Others mentioned that their culture placed great emphasis on the privacy of personal matters and therefore they would not discuss a private and stigmatizing problem with a doctor. Some also expressed concerns that a doctor might prescribe medication or institutionalize them, both of which could be harmful. In an Israeli study of late middle age and older adults, participants were asked to rate their willingness to seek a cognitive status exam in four hypothetical situations [27]. Inclination to speak with a physician about a symptom was found to be highest in response to scenarios describing a family history of Alzheimer’s disease and when the problem was seen as having more severe consequences. In another study of undergraduates from England [25], participants were given one of two vignettes that depicted symptoms indicative of either mild or moderate dementia in a person described as their mother. They were asked how they would label the problem, what they expected the problem’s duration would be, and finally whether they would seek professional or informal care for the parent, encourage the parent to seek help for themselves, do nothing for now, or do something else. The intention to seek professional help was positively associated with labeling the problem as “dementia,” (as opposed to stress or depression) as well as with regarding the symptoms as severe and with serious and possibly permanent consequences. Finally, in an online survey of adults aged 50 or older, factors associated with intentions to seek help for future cognitive impairments were examined using a vignette [26]. The vignette depicted an older adult experiencing decline in their daily functioning and exhibiting forgetfulness, repetition of conversations and events, and difficulty regulating their emotions. Participants were asked to imagine the individual in the vignette was their “future self,” and then asked to rate how likely it was that they would take each number of different actions. These included: continue to monitor one’s behavior, try to cover up one’s difficulties from family and friends, consult with family and friends, talk to one’s doctor at one’s next annual physical, make a memory evaluation appointment, do nothing. Identifying the problem as likely AD-related was associated with medical help-seeking.

In this paper, we investigate the reports from a community sample of Latinos regarding whether they would bring each of 5 different kinds of memory and thinking problems to the attention of a physician if they experienced them. Because early detection of AD relies heavily on patients or family members recognizing initial symptoms and seeking medical evaluation of them, the aim of this report was to better understand the factors influencing Latinos’ decisions to seek such help. Currently, little is known about this issue among Latinos despite the expected dramatic growth in the number of older Latinos who will be living with AD and related dementias in the coming decades and the greater likelihood older minority adults will receive a delayed or misdiagnosis than non-Hispanic Whites.

## Methods

This study was performed in line with the principles of the Declaration of Helsinki. Approval was granted by the Institutional Review Board at Columbia University Irving Medical Center [Protocol IRB-AAAR8269]. All participants in the study provided either written or electronic informed consent. All participants consented for publication of deidentified study findings.

### Design

The data for this report come from a community-based longitudinal mixed-methods study designed to assess the psychosocial, behavioral, and cognitive impacts of receiving information about future risk of developing late-onset AD. Address-based sampling of the target communities was used for recruitment. To be eligible to participate in the study, individuals had to (1) self-identify as Latino or Hispanic, (2) be 40−64 years old, (3) speak English or Spanish, (4) reside in the New York City’s northern Manhattan neighborhoods of Washington Heights, Inwood, Hamilton Heights, Central Harlem, East Harlem, Morningside Heights, Manhattanville, or North Harlem, (5) have not previously been diagnosed with AD, (6) have not previously had *APOE* genetic testing, (7) not have a family history of AD that appeared consistent with early-onset, autosomal dominant AD, and (8) respond “not at all” to the Patient Health Questionnaire (PHQ-9) suicidality item [28].

The mixed-methods study had both a quantitative (interview) and qualitative (survey) component. Participants in the qualitative component were a purposively selected subsample of the survey sample participants. They were chosen to maximize heterogeneity with respect to first-degree family history of the disease and *APOE* genotype. Efforts were also made to ensure an adequate distribution based on gender and age ranges (40–49, 50–59, 60–64). Risk information was delivered to participants by bilingual certified genetic counselors. Assessments of the impacts of receiving this information were conducted approximately 6 weeks, 9 months, and 15 months after obtaining the information. For a full description of study methods see Wetmore et al., [29].

Participants for the qualitative component were recruited from 24 August 2021–26 September 2023. Data for this report come from the first follow-up qualitative interviews conducted between 26 October 2021 and 06 June 2024 in English or Spanish based on participants’ preference and ranged from 70 to 95 minutes. Participants received a $100 gift card for each completed interview. Collection of the interview data and transcription and translation (as needed) of interview audios were done by two HIPAA compliant and encryption certified commercial firms. Quality checks of translation were performed by a bilingual research team member. An audit trail was also maintained which included keeping careful and complete documentation of changes to interview guides, codes and coding procedures providing a record of how, when and why changes to the study methodology were made. This study is reported according to the Standards for Reporting Qualitative Research (SRQR) [30].

### Data collection

The qualitative interviews were conducted by Zoom. They covered a broad range of topics in addition to those aimed at assessing the impact on participants of receiving information about their future risk for AD. One of these was the kinds of cognitive difficulties that would make a participant want to seek medical care. Specifically, interviewers asked participants about each of five specific kinds of memory or thinking problems selected from an Alzheimer’s Association-produced list of 10 early signs of Alzheimer’s disease or dementia [31]. On their website, the Alzheimer’s Association indicates that these symptoms should not be ignored but rather brought to a doctor’s attention. The interviewers introduced the question as follows: “I am going to read you a list of problems people might experience. For each one, please tell me if you experienced the problem multiple times over a period of say 2 - 3 months would it make you want to speak to a doctor to find out its cause?” The list included: (1) forgetting information you just recently were given or learned, (2) forgetting where you are or how you got there, (3) finding it hard to carry out everyday tasks such as getting confused over the correct change when shopping, (4) misplacing something and being unable to retrace your steps to find it, and (5) having trouble remembering or finding the words you are looking for to express yourself. For each symptom, participants were probed to understand the reason for their response.

### Data analysis

Interviews were transcribed verbatim and uploaded into ATLAS.ti, a software for qualitative data analysis. First, all material related to participants’ responses to the above-mentioned interview questions was extracted and a content analysis of responses to the question about the five specific symptoms was carried out [32,33]. Next, all the extractions were read to become familiar with the content and quality of the data. Open coding was used at this step of the analysis. Next, the data were sorted by symptom and carefully read again to develop inductive codes that reflected the reasons offered for endorsing or not endorsing each of the five symptoms as one they would or would not want to discuss with a doctor. The final codes and subcodes developed were reviewed by two senior team members and conceptually refined or collapsed if overlapping or redundant and then applied to all the extracted material. There was a high level of agreement between two team members once the independent application of the codes was obtained. The first author took responsibility for writing up the first draft of the results which were then reviewed and modified based on discussions and input from other team members who interacted with the data in different roles on the project.

Reflexivity was encouraged throughout the study starting with the development of interview guides, through developing codes and coding of the data, to the preparation of research reports. The entire team consisted of women from diverse disciplines, including sociology, public health, and psychology. They had previous experience with different kinds of research methods (qualitative, quantitative, community engagement) and were from diverse ethnic and cultural backgrounds. This ensured that a variety of frames of reference and perspectives were brought to bear on the data at each stage of the research. Team members challenged one another’s ideas and preconceptions, pointed out potential biases and offered alternative interpretations and the evidence for them.

## Results

### Sample characteristics

The data for this report come from 161 participants. Their mean age was 53 years, 69% were women, and a majority were Latino-Caribbeans with 50% identifying as Dominicans. Participants were generally well educated with most having more than a high school education and 55% percent of the interviews being conducted in English. Demographic characteristics are shown in Table 1.

**Table 1:** Participant Characteristics (*N* = 161).

Demographics	*n*	*%*
Age Group (in years)		
40-49	48	30
50-59	72	45
60-64	41	25
Gender		
Man	50	31
Woman	111	69
Latino Ethnic Group		
Dominican	81	50
Puerto Rican	26	16
South American	18	11
Mexican	9	6
Cuban	8	5
Central American	6	4
Other	12	8
Education		
Kindergarten to 11th grade	9	6
High school (diploma or GED)	30	19
Some college or technical training	40	25
College graduate	51	31
Graduate work	31	19
Marital Status		
Married or in a significant relationship	66	41
Single, never married	45	28
Separated, divorced or widowed	50	31
Employment Status		
Currently working (FT, PT, Self-employed)	101	63
Unemployed, looking for work	24	15
Retired	5	3
Unable to work – illness or disability	14	9
Looking after home and family	6	3
Other	11	7
Annual Household Income		
Less than $25,000	45	28
$25,000 - $49,999	47	29
$50,000 - $74,999	19	12
$75,000 - $99,999	12	7
$100,000 or more	26	16
Prefer not to say	12	7
Health Insurance		
No Insurance	10	6
Public Insurance	90	56
Private Insurance	61	38
Interview Completion Language		
English	88	55
Spanish	73	45
Family (blood relative) History (*famhx*) of AD		
First-degree family history	58	36
Other family history	49	30
No family history	54	34
Place of Birth		
Born in the US (50 states, DC, or PR)	63	39
Foreign born	98	61

### Responses to five specific kinds of memory or thinking problems

#### Forgetting where you are or how you got there.

Of the five memory or thinking problems asked about, “forgetting where you are or how you got there” was the one most frequently endorsed by participants. Virtually all participants said they would want to see a doctor if they experienced this symptom. Many were emphatic in their reply stating they “definitely” or “of course” would want to speak with a physician. Those who endorsed it said they would regard the problem as “alarming,” “a red flag,” “really serious,” or “very extreme”. Participants indicated that they would not ignore, dismiss, or delay seeing a doctor about such a problem. Many seemed to regard disorientation as a highly indicative sign of AD which is why it would be so alarming. In some cases, they had witnessed people they knew who had the disease experience this problem. Some illustrative responses to the problem “forgetting where you are or how you got there” were:

Oh, that’s very indicative…That’s when we realized something was wrong [with my mom]…She was in the house, and she would say, “What happened with my house?” I think that the loss of the sense about time and space are an indication you have Alzheimer’s. *(woman, 50–59 age group)*

That would definitely make me get help. Because that’s a clearer symptom. That would be alarming. That you don’t know how you got there or where you are, that would be very alarming. That would be a very clear sign that something is going on. *(man, 50–59 age group)*

#### Finding it hard to carry out everyday tasks.

“Finding it hard to carry out everyday tasks such as getting confused over the correct change when shopping” was another problem endorsed by most participants. Many commented that to suddenly be unable to do things they previously did if not daily at least routinely, without difficulty would certainly be a cause for considerable concern and spur them to see a doctor.

Yes [want to see a doctor]. That is something very basic—everyday tasks, the things that I do every day, [like] how to count to 100, how to make change. *(woman, 50–59 age group)*

That wouldn’t be normal if I’m given change, and I can’t determine whether it’s right or not—something I’ve done normally all my life. If it were to happen to me several times, I’d definitely go to a doctor. Of course. *(man, 60–64 age group)*

#### Forgetting information, you just recently were given or learned.

“Forgetting information, you just recently were given or learned” was a problem for which few participants said they would see a doctor about. Some of those who did endorse it, qualified their response saying that they would have to forget something very quickly to be concerned enough to seek medical attention.

If it’s recent information, let’s say something like in the past 2–3 months, I probably will not worry about it. If it’s something [like] somebody told me their name at eight o’clock in the morning, [and] I couldn’t remember by 12 noon, then I would consider that a problem. *(man, 60–64 age group)*

Not remembering what I just did. If I just did something and I don’t remember what I just did, I know I have to go see a doctor. *(woman, 50–59 age group)*

Some participants qualified their response to this symptom by differentiating between forgetting important or personally useful information and forgetting unimportant or superfluous information. While some said not recalling important or personally useful or relevant information would make them see a doctor, they indicated that not remembering information that seemed superfluous or of little personal value or relevance would not. For example, one said:

It depends on the information, right? Some information you just wanna forget anyway because there’s such an overload. If it’s important information, yes. But not just anything. *(woman, 60-64 age group)*

Another said:

Well, it depends on the information. I think that when you get older, you tend to prioritize what is relevant and what is not. We tend to [be], “Okay, I don’t care about that, give me the sensitive, the relevant information.” *(woman, 50-59 age group)*

#### Misplacing something and being unable to retrace your steps to find it.

Very few participants said “misplacing something and being unable to retrace your steps to find it” would cause them to want to speak with a physician. Most regarded this to be a very commonplace problem that everybody experiences from time to time due to circumstances like being distracted, dealing with more life stress than usual, or doing things too quickly. Therefore, they regarded this kind of problem as common and familiar to them and would not be concerned about them or feel they required a physician’s attention. As one participant said:

There have been instances where I cannot remember where I put [something], and it might just be lost until you randomly find it again. This is something that I might let go a little longer [before consulting a doctor] just because it’s something that out of all of these memory or thinking [problems asked about] thus far is kind of familiar, right? You’ve been there before, and it didn’t necessarily mean that you were having any sort of compromised mental capacity. *(man, 40-49 age group)*

Another replied:

Oh, having misplaced something. Okay. Mm. I do that sometimes actually. I could [mis]place some stuff and then I’m like, where did I put it? I don’t remember…[but] I don’t think that’s a cause of alarm… because it might be your mind is occupied on something else…That’s my thought, you know, you just did it really quickly or whatever. *(woman, 50-59 age group)*

A couple of participants who indicated this problem would cause them to see a doctor seemed to focus on the phrase “being unable to retrace your steps,” in the question which others may not have attended to enough.

Hmm, that’s tricky. Because the other day I was looking for my cell phone, and I had it on my hand. But then again, I was telling people that [it] was funny…Yes, definitely will if I cannot trace my step. *(woman, 50–59 age group)*

Of the few who thought this problem did merit speaking to a doctor, they emphasized that the problem would have to be a recurrent one.

No, unless it’s something that’s happening a lot, you know what I mean? And I don’t know what a lot is, right? I’m not gonna give it a number but, you know, um, we’ve, we’ve all misplaced something. [If it happens maybe multiple times], then yeah, I would definitely, I would definitely be concerned enough to seek help. *(woman, 50–59 age group)*

#### Having trouble remembering or finding the words you are looking for to express yourself.

“Having trouble remembering or finding the words you are looking for to express yourself” was endorsed by very few participants as a symptom for which they would seek medical care. Many said this was not a cause for concern since they had already experienced this difficulty from time to time and explained that this problem was common among Latinos who were bilingual. They reported that sometimes they could find the word they were looking for in one language (Spanish or English), but if they were talking in the other language, they might find it hard to recall the equivalent word in the other language.

Oh, that happens to me too. Sometimes I’m just talking and I’m like, oh, you know, whatever. And then, it happens to me because I speak Spanish and English. It’s like…what’s this word or whatever, something like that…I don’t think it’s really a cause of alarm me. *(woman, 50–59 age group)*

Other participants minimized the significance of this symptom in other ways, for example, by stating it was not unusual or worrisome to forget words if you did not use them regularly.

If you’re looking for a specific word that expresses more precisely what you want to say, and you can’t find it at that moment, [it’s] not because you have a problem. It’s simply not a word that you usually use, as we say in my country, “dominguera” “from Sunday/a Sunday word,” so if it’s not in your vocabulary frequently, then logically it’s easy that at a given moment when you need it, instantly, you may not remember it. *(woman, 50–59 age group)*

## Discussion

We saw considerable variability in how participants responded to the different symptoms asked about in terms of the amount of worry and urgency to seek help their comments suggested each would evoke and why they would or would not want to speak with a physician about it. Participants were most likely to say they would see a doctor if they found themselves lost and did not know where they were or how they got there. Not being able to carry out daily routines and activities was also frequently endorsed as a symptom that would prompt them to see a doctor. These symptoms seemed to be particularly concerning to participants because they strongly associated them with AD or because they would be very disruptive to their daily lives and potentially compromise their independence. Instead, we saw a tendency to want to normalize symptoms or assign a benign cause to them.

The tendency to seek benign or normalizing attributions for symptoms when they first appear is in the illness behavior literature. As defined by Mechanic, illness behavior “involves the manner in which persons monitor their bodies, define and interpret their symptoms, take remedial action, and utilize various sources of help as well as the more formal health-care system” [34]. Research has shown that when individuals experience a symptom that they recognize as not “normal” for them, they will attempt to appraise its cause and personal significance by trying to map the symptom onto an illness representations or mental prototype they already hold of different diseases or conditions [35,36]. Illness representations typically are constructed from disease stereotypes, information provided by medical professionals, media portrayals of diseases, or familiarity with a disease acquired through personal or family illness experiences. When a symptom is believed to be indicative of a disease or medical condition, it will usually be ascribed to the disease whose prototype represents the best fit [37,38]. However, when the best fit is a life-threatening, highly debilitating, or incurable disease, like AD, that can arouse great fear or threat, the individual may be motivated to arrive at a benign explanation instead or engage in minimization or accommodation to the symptom [35,39,40]. Showers & Cantor [41] have noted that while the social cognition literature emphasizes how pre-existing knowledge and disease prototypes influence symptom interpretation, people may reject the most obvious interpretation of a symptom when accepting it would threaten important personal goals, challenged strongly held important beliefs or cause significant threat or distress. This literature has largely focused on physical symptoms which makes this paper an important contribution to the literature.

Typically, only when benign explanations are no longer adequate or plausible will they consider other causes including physical illness [42]. Among our participants we observed the same tendency in response to some of the symptoms asked about that could be early signs of the disease and should be brought promptly to a doctor’s attention. For example, many attributed difficulty in finding the words to express themselves to being bilingual and which sometimes made them unable to find the word they were looking for in one language (Spanish or English) when speaking the other language. Others felt that such difficulty could sometimes be caused by not using words often, making it hard to recall them. While forgetting information recently learned or acquired was attributed by some to a disinterest in the information, choosing to forget personally trivial or uninteresting information, or being distracted, having ADHD, or as an after effect of COVID-19.

Relatively few participants said that having difficulty retaining recently acquired information required a physician’s attention. Some minimized the problem’s significance by arguing it was impossible for most people to retain all the information they acquire, or that they made little or no effort to retain information that was not personally relevant or important. A few participants even suggested they willfully forgot information they did not need or considered unimportant or uninteresting. Thus, their response to this symptom or problem seemed to depend on the nature of the information and the likely implications of forgetting it. Others, however, said that if they forgot information very quickly (e.g., within a day) or did not retain information that was important or of personal relevance, they likely would see a physician.

If one looks at responses across the 5 symptoms, rather than considering each one individually, it is possible to discern several considerations related to a symptom’s characteristics or its possible consequences that influenced whether or not participants said they would consult a physician about one or more of the problems asked about. The most important consideration associated with seeing a doctor was when the symptom or problem was part of participants’ illness representations of AD. In the case of the 5 symptoms, this most often applied to “forgetting where you are or how you got there”. Even people who do not personally know someone with AD are often familiar with the problem of “wandering” among those with the disease possibly due to media coverage of someone with AD getting lost or having seen Silver Alerts or noticed flyers in their neighborhoods requesting assistance locating a missing person with AD. It is fair to say that AD is a widely dreaded disease as it is a progressive, incurable, debilitating, and ultimately fatal. While some research has shown that when individuals experience symptoms they associate with a serious illness like cancer, they may avoid or delay seeking care out of fear [43–46], we did not find this to be the case in our study. Rather, recognizing that a symptom could likely be a sign of AD appeared to make participants inclined to want to see a doctor quickly. In the vignette study by Midden and Mast [26] cited above that looked at medical help seeking intentions in response to cognitive impairments, the authors also found that contrary to expectations based on the literature, higher threat appraisal increased the likelihood one would seek help for the symptom.

The findings also show that participants were likely to bring a symptom to a doctor’s attention if they felt it would be disruptive to their daily life, including their work life. Alonzo [47] wrote about the phenomena of “containment” in the management of somatic symptom, noting that people who are able to sustain their usual roles and activities and keep the problem of managing the symptom as a “side involvement,” typically do not seek medical care [47]. However, once this is no longer possible, and it begins to disrupt their normal activities, they typically will see a physician and enter the patient role. It appears from our data that the same pattern of behavior may exist with cognitive symptoms.

In addition, problems that participants assessed to be not normal, or at least not normal for them, were also endorsed as ones that would motivate them to see a physician. Many participants had strong convictions about certain mental competencies or abilities they possessed. They felt if those were suddenly lost or inexplicably compromised it would surely signal a serious problem. Some, for instance, felt they had a very good memory, so forgetting information quickly would be very abnormal for them. Others who saw themselves as proficient in some area like math, felt they would be very concerned if, for example, they had difficulty counting change.

Another consideration was how prevalent in the population they believed a problem, or a symptom, was. Problems they regarded as very commonplace or prevalent in the population were typically judged as not serious and therefore not requiring a physician’s attention. Participants seemed to assume that a symptom or problem that many people experienced was unlikely to be consequential. Evidence from early seminal work on the appraisal of health threats, primarily experimental in nature, has shown that individuals’ judgments about the seriousness of a health disorder (i.e., sign, symptom, disease) are influenced by what they perceive to be the prevalence of that threat [48,49]. In the current study, the symptoms that participants believed to be commonplace were trouble finding words to express oneself if bilingual and forgetting where you put something and being unable to retrace one’s steps to find it. On the other hand, many also recognized that it is not normal to suddenly realize you don’t know where you are or how you got there and that one rarely heard about this problem unless a person had AD or dementia.

In addition, if a symptom asked about was one our participants had already experienced for some extended, often very lengthy, period of time without getting meaningfully worse, they were also inclined to dismiss them as not worrisome or requiring medical attention. Some participants said they had already experienced one or more of the problems we asked about like misplacing something and not being able to retrace their steps to find it or forgetting recently obtained information. They seemed to feel reassured that if they had experienced a symptom over a long period of time and it had not gotten significantly worse, that it was unlikely to be associated with a serious problem or to a progressive disease like AD.

Another consideration that made participants unlikely to see a doctor about a symptom was if they could readily come up with a plausible benign or normalizing explanation for it. Symptom research has shown that a common reason for delay in seeking medical care is a tendency to want to normalize or minimize symptoms or assign a benign cause to them. We saw among our participants the same tendency in response to some of the symptoms asked about that could be early symptoms of AD. Some said forgetting where one put something and being unable to retrace one’s step to find it was a common occurrence in daily life and often attributed this to being distracted, doing things too fast, or being under considerable stress. Similarly, as noted above, many attributed difficulty in finding the words to express themselves to being bilingual and sometimes unable to find the word they were looking for in one language (Spanish or English) when speaking the other language. Others felt that difficulty finding the words you are looking for could sometimes be caused by not using the words often, making them hard to recall. Similarly, few thought forgetting information recently acquired or learned unless it was forgotten very quickly (e.g., within the same day or within a couple of days) would be of concern. Otherwise, this was seen as a commonplace problem that typically had a benign cause like stress or being distracted by other matters. Some minimized the problem’s significance by arguing it was impossible for most people to retain all the information they acquire, or that they made little or no effort to retain information that was not personally relevant or important. A few participants even suggested they willfully forgot information they did not need or considered unimportant or uninteresting. Others attributed forgetting information recently learned or acquired to a disinterest in the information, choosing to forget personally trivial or uninteresting information, or being distracted, having ADHD, or as an after effect of COVID-19. A few, however, said that if they forgot information very quickly after they acquired it or did not retain information that was important or of personal relevance, they likely would want to see a physician. Thus, their response to this symptom or problem seemed to depend on the nature of the information and the likely implications for them to forget it.

Finally, if participants felt that the problem asked about occurred among people of all ages, they also tended to dismiss it as not significant or needing medical attention. Presumably because Alzheimer’s is regarded as a disease of the elderly, participants commonly mentioned seeing some of the problems we asked about in many young people or having dealt with the problem since they were much younger as reasons for not feeling the need to consult a physician. Forgetting where one put something and being unable to retrace one’s steps to find it for example, was a problem most participants felt was common in all age groups, even children and young adults. Others said they had always been forgetful so that symptom did not worry them.

This report makes a unique contribution to the literature by focusing on Latinos, an important population given the expected dramatic increases in AD among Latino older adults in the coming decades and the greater likelihood of minority older adults experiencing a missed or delayed diagnosis for dementia than their non-Hispanic White peers [10–13]. Further because it investigated a variety of symptoms rather than, as in most previous reports, focusing on a single (usual memory) problem it was possible to identify considerations that influenced participants’ inclination to seek or not seek medical consultation that cut across a variety of symptoms. This is important in trying to ensure a timely diagnosis. The findings also support the need for greater education among Latino about possible early signs of AD and the benefits to seeking an early diagnosis.

However, there are also limitations to this report that should be noted. Perhaps the most significant limitation is that the questions were hypothetical and asked about participants’ expected responses to memory or thinking problems they did not currently have but could potentially develop in the future. The extent to which their answers reflect what they would actually do if they were to develop such symptoms at a later time in a different context is unknown. Another limitation is that we know people often discuss their symptoms with family and friends who can influence their decisions about what action to take or even try to provide informal care which can delay seeking medical care. It was not possible for us to examine the influence of family or friends because of the hypothetical nature of the questions. Finally, we cannot generalize from our data to all Latinos because we are using a purposive sample rather than a representative one. In addition, our participants were primarily Caribbean Latinos and predominantly Dominican so they may not be representative of all Latinos. However, given the dearth of information about Latinos and help-seeking for memory and thinking problems, this report offers novel insight into Latinos’ perceptions of cognitive symptoms which can be further investigated in a more representative sample.

## Supporting information

S1 File Additional Quotations: Identified reasons for bringing each of the five thinking or memory problems to a doctor’s attention. (DOCX)
