# Supplementary material for: Memory and thinking problems that aging Latinos in New York City would bring to a doctor’s attention
Source: PLoS One. 2026 May 20;21(5):e0349635. doi: 10.1371/journal.pone.0349635 (PMC13189322; doi:10.1371/journal.pone.0349635)
Supplement: S1 File — (DOCX) [file pone.0349635.s001.docx]

**S1 File.** **Additional Quotations: Identified reasons for bringing each of the five memory or thinking problems to a doctor’s attention.**

| **Themes** | **Quotations** | |
| --- | --- | --- |
| **Forgetting where you are or how you got there** | (1) | For example, getting lost if you go out, and not knowing where you are and you have to call, “Oh, son, come and get me”. That's a difficult problem. Or [not knowing how to] go back to the house. That is dangerous! *(woman, 50-59 age group)* |
|  | (2) | Oh, [forgetting where you are or how you got there], that's a good one. That's a good one. That's a very serious one. That is one that God forbid I ever experience. Oh, my goodness. Oh, boy. I will take care of it immediately. To forget -- to forget where you are -- to forget where you -- Oh, no, that is really serious. *(woman, 60-64 age group)* |
|  | (3) | Oh, definitely, sure. Again, because that [forgetting where you are or how you got there] means that, uh, that I'm losing control of, um, myself as a person not knowing where I am. That would be very concerning. *(man, 60-64 age group)* |
|  | (4) | Yeah. Maybe that would be a little scary. Forgetting where I was and how I got there. Mm. Yeah, maybe that would be, yes, that would be cause of alarm. Yes. *(woman, 50-59 age group)* |
|  | (5) | Oh, heck, yeah, I definitely would be talking to a doctor. Yes, because I would be really scared. I would be - I would start thinking about my aunt, you know, she, she was a smoker and she left the house while under my care and walked two miles in a nightgown. … They found her like two miles away. So she doesn't know, she had no sense, because of her Alzheimer's. She had no sense of time. She didn't understand where she was. She just knew that she had a need and she wanted to fulfill that need. … So definitely if I see where I didn't know where I was at or I lost track of something like that I would definitely be going to seek some help because I see what that can do to you. She could have been raped, she could have been killed, abducted, you know, it, it was just scary what, what she did, you know. So, yeah, definitely. I would definitely be looking at that. *(woman, 60-64 age group)* |
| **Finding it hard to carry out everyday tasks** | (1) | Yeah, because I'm very obsessive about that [the correct change when shopping] to the point of, like, giving people two extra pennies or something in the -- in the [laundromat--]. And if I couldn't even figure out what I was getting back, I would be scared. I would definitely be talking to someone. *(man, 50-59 age group)* |
|  | (2) | Um, that [finding it hard to carry out everyday tasks such as getting confused over the correct change when shopping] would worry me. Yes I'd have my concerns. Yeah, it would like, it would probably make me go to the doctor. It has to take a lot to make me go to a doctor, to be honest with you. Because I've never been too much of a doctor person or hospitals or anything like that. *(woman, 50-59 age group)* |
|  | (3) | Oh, yeah, for sure. I mean if you, if you can't count your money, you know, [laughs] that's a, that's a problem for sure. *(man, 40-49 age group)* |
|  | (4) | If it happens enough-- if it happens once, maybe not. If it happened already like three times, yes.. . . because this is something that has never happened before, you know? Um, I mean, can you, at a rush you're buying something and, you know, and, you know, make a mistake, yes, that could happen to anybody. But if it's something that's occurring and it occurred again and again and it's something that wasn't something that's happened before, then yeah, I would wanna tell a doctor for sure. *(woman, 50-59 age group)* |
| **Forgetting information, you just recently were given or learned** | (1) | Um, maybe. Sometimes, that does happen to me, but-- but I mean, it's either very recent information or I don't-- I'm just not interested in it, so, I might forget it quickly. I associate it with that. *(man, 40-49 age group)* |
|  | (2) | If it’s an important piece of information that I forget, I would. *(woman, 40-49 age group)* |
|  | (3) | Not necessarily, because imagine, one reads so much and searches so many things, especially with... one is bombarded with cyber information. It's a lot. *(woman, 50-59 age group)* |
|  | (4) | No, because I know that for me to learn something, it takes at least 21 days to learn it and if it's not related to my daily life and it's not, I'm not gonna be using it, it's, it's, it's gonna be gone because I don't need it. But if it's something that is related to what I'm studying or like I'm gonna be working with, you know, it's, it's gonna be there. … it takes at least 21 days to learn a new skill and to become a habit in your, in your, in your daily life. And I don't, that wouldn't, unless it's like something that I, I don't know, I don't think so. No, that wouldn't worry me. That wouldn't, that wouldn't make me go to the doctor. No. *(woman, 40-49 age group)* |
| **Misplacing something and being unable to retrace your steps to find it** | (1) | That’s happened to me before and then I come down and then retrace it again and then I find it. Yeah, misplacing is something that’s, you know, often. I mean, it’s normal. *(woman, 50-59 age group)* |
|  | (2) | Uh, that not much because that, that, that happens time to time, you know, and I see people [laughter] younger than me that, you know, will misplace something oh where is it or, um... *(woman, 60-64 age group)* |
|  | (3) | It depends. Because sometimes it’s already happened to me that you can’t find the key. It does come easy to me and what I do is I go backwards, like we say, okay, let me go back to the beginning, I was here, go back on all the things that I did. But I think that’s something that almost always happens to us in the stress of life, forgetting things. And in my case, at least, it’s always just a matter of stopping, breathing and going back. So, I don’t think that would be an alert in my life to say, I’m going to the doctor, because I think that with the stress of life, with children, work, the house... I mean, I think that is something that happens too often. *(woman, 40-49 age group)* |
| **Having trouble remembering or finding the words you are looking for to express yourself** | (1) | No, that happens to me. Sorry and then, as I said, it’s not that my English is perfect, but sometimes I look for a word in Spanish and I, because I’m not use, yes, I do use it, but not as a 100 percent of my time. There are some words in Spanish that I have stopped using, like [slang term], how do you say that…? *(woman, 50-59 age group)* |
|  | (2) | That's a given for me. That happens to me a lot. [laughs] You know, sometimes I wanna, I wanna say something and I kinda, kinda look up to figure out what's the word I'm looking for but that's just, that's just me. I don't think it has anything to do with any disease, that's just me. *(woman, 50-59 age group)* |
